# Assessing the hazard of death, cardiac tamponade, and pericardial constriction among HIV and tuberculosis pericarditis patients using the extended Cox‐hazard model: Intervention study

**DOI:** 10.1002/hsr2.1892

**Published:** 2024-02-14

**Authors:** Abdul‐Karim Iddrisu, Dominic Otoo, Afa Kwasi, Freedom Gumedze

**Affiliations:** ^1^ Department of Mathematics and Statistics University of Energy and Natural Resources Sunyani Ghana; ^2^ Department of Statistical Sciences University of Cape Town Rondebosch South Africa

**Keywords:** cardiac tamponade, composite outcome, constriction, extended or time‐dependent Cox proportional hazard model, prednisolone, tuberculosis pericarditis

## Abstract

**Background and Aims:**

Tuberculous (TB) pericarditis (TBP), a TB of the heart, is linked to significant morbidity and mortality rates. Administering glucocorticoid therapy to individuals with TBP might enhance overall results and lower the likelihood of fatality. However, the actual clinical effectiveness of supplementary glucocorticoids remains uncertain. This study specifically evaluated the effects of prednisolone, prednisolone‐antiretroviral therapy (ART) interaction, and other potential risk factors in reducing the hazard of the composite outcome, death, cardiac tamponade, and constriction, among TBP and human immunodeficiency virus (HIV) patients.

**Methods:**

The data used in this study were obtained from the investigation of the Management of Pericarditis trial, a multicentre international randomized double‐blind placebo‐controlled 2×2 factorial study that investigated the effects of two TB treatments, prednisolone and *Mycobacterium indicus pranii*  immunotherapy in patients with TBP in Africa. This study used a sample size of 587 TBP and HIV‐positive patients randomized into prednisolone and its corresponding placebo arm. We used the extended Cox‐proportional hazard model to evaluate the effects of the covariates on the hazard of the survival outcomes. Models fitting and parameter estimation were carried out using R version 4.3.1.

**Results:**

Prednisolone reduces the hazard of composite outcome (hazrad ratio [HR] = 0.32, 95% confidence interval [CI] = 0.19,0.54, *p* < 0.001), cardiac tamponade (HR = 0.14, 95% CI = 0.05, 0.42, *p* < 0.001) and constriction (HR = 0.81, 95% CI = 0.41, 1.61, *p* = 0.55). However, prednisolone increases the hazard of death (HR = 1.58, 95% CI = 1.11, 2.24, *p* = 0.01). Consistent usage of ART reduces the hazard of composite outcome, death, and constriction but insignificantly increased the hazard of cardiac tamponade.

**Conclusion:**

The study offers valuable insights into how prednisolone impact the hazard of different outcomes in patients with TBP and HIV. The findings hold potential clinical significance, particularly in guiding treatment decisions and devising strategies to enhance outcomes in this specific patient group. However, there are concerns about prednisolone potentially increasing the risk of death due to HIV‐related death.

## 1 INTRODUCTION

Tuberculous (TB) pericarditis (TBP), a TB of the heart, is widespread in sub‐Saharan Africa and specific regions of Asia, often resulting in conditions such as pericardial effusion, cardiac tamponade, and constrictive pericarditis.^1^, ^2^, ^3^ It is important to highlight that individuals with TBP frequently experience concomitant human immunodeficiency virus (HIV) infection. Even with anti‐TB chemotherapy, nearly half of individuals affected by TBP face the risk of death or disability.^4^ At the 6‐month mark, mortality rates can escalate to 26%, but for those with acquired immunodeficiency syndrome, this figure climbs even higher, reaching approximately 40%.^5^ Enhancing the outcomes of TBP may involve mitigating inflammatory responses to reduce cardiac tamponade and pericardial constriction.^6^ However, there is uncertainty regarding the safety and efficacy of adjunctive immunomodulation using corticosteroids and *Mycobacterium indicus pranii* (M.w) immunotherapy in lowering mortality and morbidity.^7^, ^8^, ^9^

Conflicting evidence concerning this combined therapy has resulted in divergent recommendations.^10^ For example, there is a concern that corticosteroids may elevate the risk of opportunistic infections and cancers in HIV‐infected patients.^11^ Although adjunctive steroid therapy has shown promise, the evidence remains inconclusive.^11^ Conversely, preliminary findings suggest that repeated doses of M.w might alleviate inflammation in extrapulmonary TB and boost CD4 cell count in individuals with HIV.^10^ However, these initial observations require validation through a large randomized trial with mortality as the primary endpoint.^11^ Furthermore, in a meta‐analysis of randomized controlled trials investigating glucocorticoid therapy for TBP, there was a reduction in mortality, but the results did not reach statistical significance.^8^, ^9^, ^11^ The limitations of the study included a small number of events and a restricted number of patients included in the analysis.^8^, ^9^, ^11^ On the other hand, a meta‐analysis of all trials examining adjunctive glucocorticoid therapy for various forms of TB also suggested a potential decrease in mortality.^12^

Moreover, conflicting recommendations are present in international guidelines^7^, ^13^ regarding the use of adjunctive glucocorticoid therapy in patients with TBP due to various factors.^7^, ^13^ Although glucocorticoids have demonstrated potential in reducing mortality among TB patients, concerns persist about an elevated risk of cancer in HIV‐infected individuals treated with glucocorticoids.^7^, ^13^ In addition, there is limited evidence concerning the effects of adjunctive glucocorticoid therapy for TB in HIV‐infected patients, adding to the uncertainty surrounding its role in treating TBP.^7^, ^13^ To address this ongoing debate, Mayosi et al.^10^, ^11^ initiated the Investigation of the Management of Pericarditis (IMPI) trial. Their hypothesis for the trial posited that incorporating prednisolone as an adjunctive treatment would confer a comprehensive advantage for these patients.^11^

Preliminary findings suggest that the administration of repeated doses of intradermal heat‐killed *Mycobacterium indicus pranii* (formerly known as *Mycobacterium w*) immunotherapy holds promise in reducing inflammation associated with TB and increasing CD4 + T‐cell count in individuals with HIV infection.^14^, ^15^, ^16^ M.w, a nonpathogenic, saprophytic, and rapidly growing atypical mycobacterium species, has demonstrated clinical benefits when administered as a heat‐killed intradermal formulation in leprosy patients and potentially in those with pulmonary TB and HIV infection.^14^, ^17^, ^18^, ^19^, ^20^ Building on this, Mayosi et al.^10^, ^11^ hypothesized that intradermal M.w could effectively suppress inflammation and its consequences in patients with TBP. To investigate this hypothesis, the IMPI trial was conducted to assess the efficacy and safety of adjunctive prednisolone and M.w in African patients with TBP. The primary objective of the IMPI trial was to evaluate the efficacy and safety of prednisolone and M.w in reducing the combined occurrence of death, constriction, or cardiac tamponade requiring pericardial drainage in a cohort of 1400 patients with TB pericardial effusion.^10^, ^11^

Mayosi et al.^11^ determined that there was no interaction between prednisolone and M.w. Consequently, separate analyses were conducted for the prednisolone arm versus its placebo arm and the M.w arm versus its placebo arm.^11^ The findings from the IMPI trial indicate no significant differences in the primary outcome between patients who received prednisolone and those who received placebo, or between those who received M.w and those who received placebo.^11^ However, in comparison to the placebo group, prednisolone therapy exhibited a notable reduction in the occurrence of constrictive pericarditis and hospitalization. Additionally, both prednisolone and M.w, when compared to the placebo, were associated with a significant increase in the incidence of cancer, primarily attributed to a rise in HIV‐associated cancer.^11^

Also, the impact of IMPI trial medications and their potential interactions with antiretroviral therapy (ART) on changes in CD4 count remained uncertain. Identifying a significant interaction effect on CD4 count changes could offer additional insights into the effects of medications employed in the IMPI trial on the composite outcome. To address this inquiry, Iddrisu and Gumedze^21^, ^22^, ^23^, ^24^ undertook an investigation into the effects of prednisolone, the interaction between prednisolone and ART, and other predictors on changes in CD4 count over time.^11^ To ensure the reporting of valid statistical inferences despite missing values in CD4 count measurements, these researchers employed various sensitivity analysis approaches for handling the missing data.^21^, ^22^, ^23^, ^24^

Mayosi et al.^11^ suggest that the interventions were ineffective due to the relatively low proportion of trial participants diagnosed with TBP. However, their results showed that patients with definite and probable TB suggests that prednisolone is anticipated to confer a protective effect against the composite outcome, cardiac tamponade, and constriction.

In this study, we aimed assess effect of TB medication (prednisolone) in reducing the time‐to‐first occurrence of (1) composite outcome of death, cardiac tamponade, or pericardial constriction, (2) death, (3) cardiac tamponade, and (4) pericardial constriction (constriction), among only HIV‐positive and TBP patients using the extended or time‐dependent Cox‐proportional hazard (PH) model.^25^, ^26^, ^27^, ^28^, ^29^, ^30^ These models are applied to data on patients with TBP that were randomized into the prednisolone and its corresponding placebo arm.^2^, ^10^, ^11^ This study also adjusted for the effects of the following covariates: ART (ever on ART during the study vs. never on ART during the study), prednisolone–ART interaction, age above 46 versus at 46 and below, baseline ART (on ART at study entry vs. not on ART at study entry) and gender (male vs. female) on the survival outcomes. The key hypotheses that derive this study are (1) the hazard of the event significantly differ between prednisolone and placebo arms, (2) patients who received ART at each visit is associated with reduced hazard of the survival outcome, and (3) there is no interaction between prednisolone and ART treatments, among the HIV positive patients only.

## 2 METHODS

2.1

In this section, we discuss the Cox‐PH model for survival data^31^, ^32^, ^33^, ^34^ as well as the extended or time‐dependent Cox‐PH model for handling covariates that violates the assumption of constant covariate effect on the hazard over time.^35^, ^36^, ^37^

#### 2.1.1 Cox‐PH model

The Cox‐PH model^32^ is used to estimate the effects of covariates on the hazard of the event. Of course, there are different models for time‐to‐event data.^38^ However, the most popularly used time‐to‐event model is the semiparametric Cox‐PH model.^32^ In the Cox‐PH model, the baseline hazard function is estimated nonparametrically. If for any reason, there is the need for baseline hazard function to assume a parametric form (such as the Weibull, Gamma, or Exponential), a parametric model can be specified. However, it is a common practice for researchers to ignore the exact form of the hazard function.^25^ Fox and Weisberg^39^ study revealed that the Cox‐PH model approximate well unknown parametric hazard functions.^25^

Consider *p* risk factors or explanatory variables, $x_{1},x_{2},\ldots ,x_{p},\beta_{0},\ldots ,\beta_{p}$ are the regression coefficient representing the effects of the covariates on the hazard of the event, and $h_{0}$‐baseline hazard as the hazard function for an individual for whom the values of all the explanatory variables are zero. the form of Cox's PHs model is

(1) $$h_{i}\left(j\right)=h_{0}\left(j\right)\text{exp}\left(\beta_{1}x_{i1}+\beta_{2}x_{i2}+\ldots +\beta_{p}x_{\mathrm{ip}}\right),$$

where $\eta_{i}=\beta_{1}x_{i1}+\beta_{2}x_{i2}+\ldots +\beta_{p}x_{\mathrm{ip}}$ is the linear component of the model, also called the “risk score” or “prognostic index.” The model can alternatively be expressed as

(2) $$\log\frac{h_{i}\left(j\right)}{h_{0}\left(j\right)}=\beta_{1}x_{i1}+\beta_{2}x_{i2}+\ldots +\beta_{p}x_{\mathrm{ip}}.$$

This means that the Cox‐PH model may also be viewed as a linear model for the logarithm of the hazard ratio.

It is important to noted that we have not made any assumptions in terms of the actual form of the baseline hazard function $h_{0}(j)$. In fact, estimation of the β‐coefficients happen independently of $h_{0}(j)$. However, sometimes we need to estimate $h_{0}(j)$ itself. Just like in the generalized linear model, covariates in the linear component of the PHs model can be of different types such as continuous variables, for example, age or blood pressure, weight, height, hemoglobin, or binary factor variables such as gender = male/female, infection status or factor variables with more than two levels, for example, treatment groups. These latter types of factor variables can be included in the linear part of the model as linear combinations of binary dummy variables, just as we did for linear and logistic regression.

To fit the Cox‐PH model, we need to estimate (1) the unknown vector of *β* coefficients in the linear component of the model and (2) the baseline hazard function $h_{0}(j)$. These two components of the model can be estimated separately, and in fact, the *β*'s are estimated first and these estimates are then used to obtain an estimate of $h_{0}(j)$ to make inferences about the effects of the p explanatory variables $x_{1},x_{2},\ldots ,x_{p}$ on the relative hazard $\frac{h_{i}(j)}{h_{0}(j)}$; we do not need an estimate of $h_{0}(j)$. The *β*‐coefficients in the Cox‐PH model can be estimated using the method of maximum likelihood. The maximization of the log‐likelihood function is accomplished using the Newton–Raphson procedure.

#### 2.1.2 Assessing the assumption of the Cox‐PH

The premise of the PH assumption is that the hazard function, also known as the hazard ratio, for different groups, must maintain proportionality over time. That is, the effect of the different covariates over time remains constant. This implies that the hazard ratio remains consistent throughout the observation period. It is crucial to verify these assumptions before regularly applying Cox‐PH regression analysis. Several tests are accessible for examining these assumptions.^40^ These include (1) examining the Kaplan–Meier; Kaplan–Meier curves of the different groups crossing implies high probability of violation and if the Kaplan–Meier curve of one group drops down, while the other plateaus also indicate potential violation and (2) examination of Scaled Schoenfeld residuals which are statistical tests and graphical displays which check the PH assumption. When covariate is found to violate the constant covariate effect assumption, an extended or time‐depended Cox‐PH model is used.^35^, ^36^, ^37^

#### 2.1.3 Extended Cox‐PH model

When covariate(s) in the Cox‐PH model violate the constant effect of covariate over time, we used the extended or time‐dependent Cox‐PH model.^35^, ^36^, ^37^, ^41^ Assume one wants to assess whether a variable X exhibits a changing impact on the hazard of the event over time. To investigate this, a time‐related variable is constructed by combining the predictor *X*, which can be continuous or categorical, with a time function$t\left(f(t)=t,t^{2},\log(t),\sqrt{\left(t\right)},\ldots \right)$. By adding this interaction term to the model, the hazard function (1) then becomes

$$\begin{array}{cc} h_{x}(t)=h_{0}(t)\text{exp}(\beta X+\gamma \mathrm{Xf}(t))=h_{0}(t)\text{exp}\left(\sum_{p=1}^{P}\beta_{p}X_{p}+\sum_{p=1}^{P}\gamma_{p}X_{p}f(t)\right), & \end{array}$$

where *y* is the effect of *X* as time f(t) progresses. Consequently, the hazard ratio can be expressed as $\text{HR}(t)=h_{\left(x+1\right)}(t)/h_{x}(t)$, representing the change in hazard rates due to a one‐unit increase in the variable *X*. This ratio varies with time as dictated by the function f(t). When γ>0, the hazard ratio increases, and when (γ<0), the hazard ratio decreases as time progresses. This means that covariates, under Table 1, that violated the Cox‐PH model assumption must have their interaction with time terms included in the model (4). An alternative technique for incorporating time‐varying coefficients involves employing a step function, such as $f(t)=I\left(t\geq t_{0}\right)$, where $t_{0}$ denotes a threshold value. This approach involves segmenting the analysis period into multiple intervals, with the Cox‐PH being stratified across these time segments. Consequently, the impact of consistent baseline covariates fluctuates in strength over time, allowing for investigation through time‐based stratification. Either including the interaction term or stratifying the time into intervals would address the model violation. In this study, we address the violation using the step function approach where the time is stratified into group of time intervals.

**Table 1:** Testing the constant covariate effect on hazard of the composite outcome, death, cardiac tamponade, and constriction.

Composite outcome				Death		
Variable	*χ* ^2^	*df*	*p* Value	*χ* ^2^	*df*	*p* Value
Prednisolone	20.6698	1	<0.001	1.13	1	0.29
*ART*	1.5728	1	0.21	7.77	1	0.005
Prednisolone × ART	7.4845	1	0.006	4.27	1	0.04
Age	0.0926	1	0.76	1.43	1	0.23
*bonarvs*	0.9054	1	0.64	5.76	1	0.06
*gender*	1.3047	1	0.25	1.66	1	0.20
Global	27.3582	6	0.00029	15.66	6	0.0284

Cardiac tamponade				Constriction		
Prednisolone	23.724	1	<0.001	14.24	1	<0.001
*ART*	1.343	1	0.25	2.27	1	0.13
Prednisolone × ART	7.824	1	0.005	2.56	1	0.11
Age	0.145	1	0.70	2.13	1	0.14
*bonarvs*	1.742	1	0.19	2.81	1	0.09
*gender*	0.557	1	0.46	17.41	1	<0.001
Global	31.483	6	<0.001	37.30	6	<0.001

Abbreviation: ART, antiretroviral therapy.

## 3 RESULTS

This section describes IMPI trial with focus on and survival outcomes; death, cardiac tamponade, pericardial constriction, or composite outcome of death, cardiac tamponade, or pericardial constriction. We then applied the Cox‐PH model to the survival outcomes adjusting for the effects of prednisolone, ever on ART during the study, prednisolone‐ART interaction, age at trial entry, gender, and on ART at study entry. In this study, four Cox‐PH models are fitted. These include model for the composite outcome, death, cardiac tamponade, and constriction. For each specific model, an evaluation is conducted to detect any potential violations of the constant covariate effect assumption, aiming to rectify such deviations.

### 3.1 Description of IMPI trial data

The data used in this study are obtained from the IMPI trial.^10^, ^11^ The IMPI trial was a multicentre international randomized doubled‐blind placebo‐controlled 2×2 factorial study.^21^, ^22^, ^23^ Individuals could participate in the trial if they were 18 years old or above, had a confirmed pericardial effusion through echocardiography, exhibited evidence of definite or probable TBP based on the definition provided in reference,^11^ and had initiated anti‐TB treatment within 1 week before enrollment. Exclusions from the trial applied to those with an identifiable alternative cause of pericardial disease, recent use of glucocorticoids within the past month, known hypersensitivity or allergy to the M. indicus pranii preparation, or pregnancy.^11^ The IMPI trial investigated the effects of two TB treatments, prednisolone and *Mycobacterium indicus pranii* (*M. indicus pranii*) immunotherapy, in patients with TBP patients in Africa. TBP is a TB that occurs in the heart and is an important complication of TB, which diagnosis can be difficult to establish and is often delayed or missed, resulting in late complications such as composite outcome (death, cardiac tamponade, or pericardial constriction) and increased mortality.^42^ Patient who met the inclusion criteria, defined in the IMPI trial study, were randomized into four treatment arms. Randomized patients received the combination of either $M^{+}P^{+}$ or $M^{+}P^{-}$ or $M^{-}P^{+}$ or $M^{-}P^{-}$, where $M^{+}$and $M^{-}$denote the *M. indicus pranii* and its corresponding placebo arm and $P^{+}$and $P^{-}$denote prednisolone and its corresponding placebo arm, respectively. Mayosi et al.^11^ studies revealed that countries with limited resources coupled with concomitant epidemics of HIV infection are characterized by high mortality related to TBP. Mayosi et al.^3^, ^9^ revealed that there is no interaction between the two TB treatments, $M^{+}$and $P^{+}$and hence $M^{+}$and its corresponding placebo arm $M^{-}$ as well as $P^{+}$and its corresponding placebo $\text{arm}P^{-}$were analyzed separately.^3^, ^9^

In the IMPI trial, a sample size of 1400 patients with definite probable TB pericardial effusion, from nine African countries in 19 centres were enrolled in the 4‐year trial. Patients who met the inclusion criteria were randomized to receive oral pill prednisolone for 6 weeks and *M. indicus pranii* or placebo for 3 months. In general, after randomization at baseline Week 0, patients were followed up at Weeks 2, 4, and months 3 and 6 during the intervention period and 6‐monthly thereafter for up to 4 years.^3^, ^9^ Randomized patients discontinued *M. indicus pranii* treatment after 3 months due to side effect.^3^, ^9^

The IMPI trial was conducted from January 2009 through February 2014 at 19 hospitals in eight African countries.^10^, ^11^ For the comparison of prednisolone with placebo, 706 patients were assigned to receive prednisolone and 694 to receive placebo. For the comparison of *M. indicus pranii* with placebo, 625 were assigned to receive *M. indicus pranii* and 625 to receive placebo. The trial was powered for a rate of nonadherence of 10% in the active‐treatment groups. This rate was almost achieved (with nonadherence rate of 11%) in the prednisolone group and nonadherence rate was higher in the *M. indicus pranii* group (21%), owing mainly to injection‐site side effects.^11^ Also, approximately 18% deaths occurred due to TBP‐related (4.2%), TB‐related other than pericardial (3.3%), HIV related (1.3%), cardiovascular‐related (1%), unknown (3.5%).^10^, ^11^

The IMPI trial aim was to assess the effectiveness and safety of oral pill prednisolone and M.w injection in reducing the time to first occurrence of the primary composite outcome of death, pericardial constriction, or cardiac tamponade requiring pericardial drainage in patients with TB pericardial effusion.^3^, ^9^ These authors considered survival analysis on both patients with and without HIV. In this study, we investigated the effect of prednisolone in reducing the time‐to‐first occurrence of the composite outcome, death, cardiac tamponade and constriction. The sample size, n=587, consist of only TBP patients who are HIV+. Specifically, the aim of this study is to assess the effect of trial medication (prednisolone) on hazard of composite outcome, death, cardiac tamponade, and pericardial constriction among this patient population.

#### 3.1.1 Description of the survival outcomes

We have stated that the time‐to‐event of interest in this study are death (death), cardiac tamponade (*cardtamp*), pericardial constriction (*cons*), or composite outcome (*comp*). The survival outcomes; death takes the value of 1 if death occurs and 0 if censored, *cardtamp* takes the value of 1 if cardiac tamponade occurs and 0 if censored, *cons* takes the value of 1 if constriction occurs and 0 if censored, and *comp* takes value of 1 if death, cardiac tamponade or constriction occurs and 0 if censored. The treatment arms variable *pred* (takes the value of 1 if patient is randomized to prednisolone arm and 0 if placebo arm).

#### 3.1.2 Covariates in both longitudinal and survival models

Apart from prednisolone, we also examined the relationship between survival outcomes and the following covariates: *gender* (takes values of1 for male, 0 for female), *ART* (takes values 1 if ever on ART during the study, 0 if never on ART during the study), *bonavrs* at study entry (takes 0 if not on ART at study entry, and 1 if on ART at study entry), and *agegrp* (takes values 1 if age >46, 0 if age ≤46) and *pred‐ART* interaction (*predart*).

#### 3.1.3 Kaplan–Meier curves and log‐rank test

**Figure 1:**
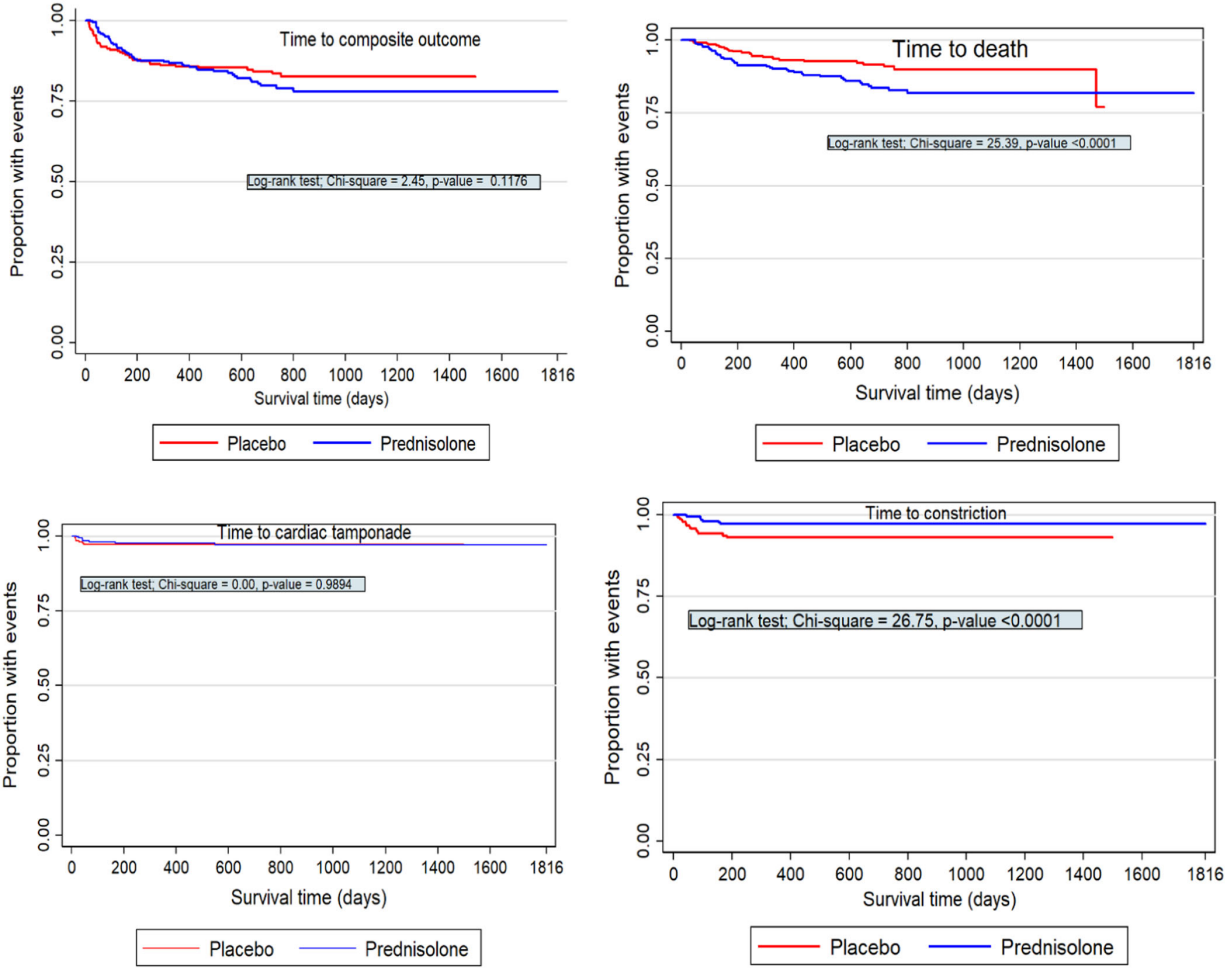
Kaplan–Meier curves and log‐rank estimates for composite outcome (top‐left panel), death (top‐right panel), cardiac tamponade (bottom‐left panel), or pericardial constriction (bottom‐right), by prednisolone versus placebo arms.

We estimate the proportion of the survival outcomes in both prednisolone and placebo treatment arms using the Kaplan–Meier method and then assess significance difference in the incidence of experiencing the event in both arms using the log‐rank test.^31^, ^38^, ^43^, ^44^ The Kaplan–Meier curves and the log‐rank results are presented in Figure 1.

The results in the top‐left panel of Figure 1 indicate that there is no significant difference $(\chi_{1,0.025}^{2}=$ 2.45, *p* =0.118) in the proportion with composite outcome between the prednisolone and placebo arms. The results in bottom left panel of Figure 1 also indicate that there is no significant difference $(\chi_{1,0.025}^{2}=0.00,$ *p* =0.989) in the risk of cardiac tamponade between the prednisolone and placebo arms. On the other hand, the bottom‐left and right panels showed significant difference $(\chi_{1,0.025}^{2}=25.39$, *p* <0.001) and $(\chi_{1,0.025}^{2}=26.75,$ *p* <0.001) in the risk of death and constriction, respectively, between the prednisolone and placebo arms.

The association between the other covariates and survival outcomes were also assessed using the Kaplan–Meier curves and the log‐rank test. The results showed significant association between composite outcome versus gender, death versus gender, and constriction versus gender, and cardiac tamponade is not associated with gender. On the other hand, composite outcome, death, and cardiac tamponade were associated with ART, whereas constriction was not associated with ART. Also, the Kaplan–Meier curves and log‐rank test statistics showed that death or cardiac tamponade is associated with ART at study entry with the exception of constriction and the composite outcome. The results also revealed that composite outcome or death was associated with age but constriction or cardiac tamponade were not associated with death.

### 3.2 Results from the Cox‐PH model

In this section, we fit the Cox‐PH model (1)^25^, ^32^, ^39^ to the survival outcomes. The Cox‐PH model adjusted for covariates effects on the hazard of the event. The Cox‐PH model (1) to be fitted has the form

(3) $$\begin{array}{cc} h_{i}\left(j\right)= & h_{0}\left(j\right)\exp\left(\beta_{1}{\text{pred}}_{i}+\beta_{2}ART_{ij}+\beta_{3}{\text{predart}}_{ij}+\beta_{4}agegrp_{i}\right)\\ & \times \;\exp\left(\beta_{5}{\text{bonarvs}}_{i}+\beta_{6}{\text{gender}}_{i}\right) \end{array}.$$

The Cox‐PH model (3) was fitted in R version 4.3.1, for each of the survival outcomes, death, cardiac tamponade, constriction, and composite outcome, using **coxph** function from survival package.^45^, ^46^ We have attached a file containing annotated R code for all analyses conducted in this paper.

#### 3.2.1 Checking violation of the constant covariate (Cox‐PH) assumption

Statistical test of the Cox‐PH model assumption is achieved by using the Scaled Schoenfeld residuals via *cox.zph* function. The results from this statistical test are presented in Table 1. Using the results in Table 1, this assumption is considered violated when the *p* value linked to a specific covariate is below 0.05. Table 1 reveals that the Cox‐PH assumption is breached by the prednisolone variable (*pred*) in relation to the composite outcome, cardiac tamponade, and constriction. Additionally, the interaction between prednisolone and ART (*predart*) violates the Cox‐PH assumption in cases involving the composite outcome, death, and cardiac tamponade. Furthermore, the *gender* variable deviates from the Cox‐PH model assumption concerning constriction.

**Figure 2:**
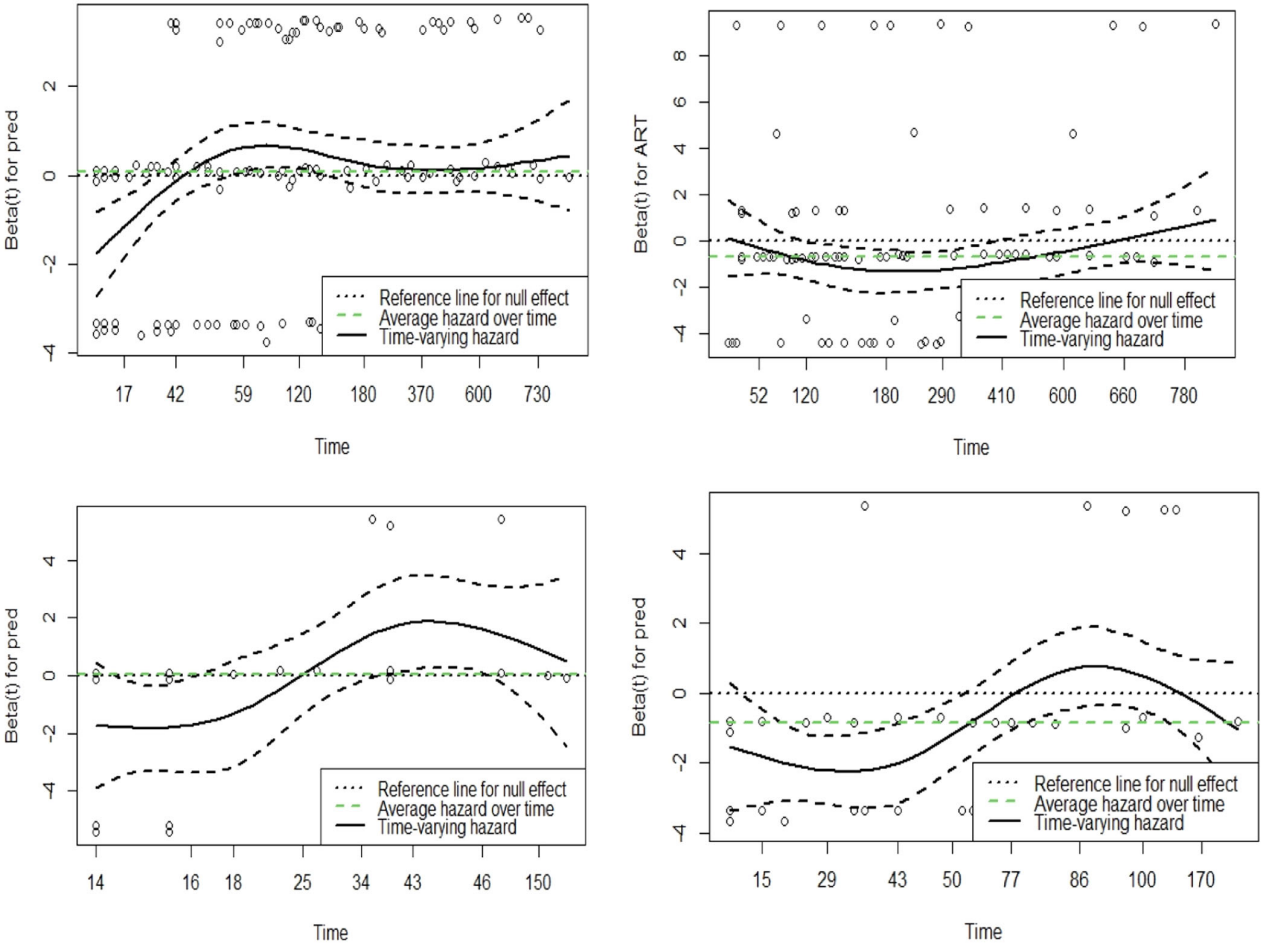
The effects of covariates; pred on composite outcome (top‐left panel), cardiac tamponade (bottom‐left panel) and constriction (bottom‐right panel), and effect of *ART* on death (top‐right panel) varies over time.

The main effects such as *pred* and *ART* variables that violated the constant covariate effect over time assumption are displayed graphically in Figure 2. Figure 2 showed that *pred* is a time‐varying coefficient for under the Cox‐PH model for composite outcome, cardiac tamponade, and constriction and *ART* is a time‐varying covariate under the Cox‐PH model for death. The *pred* and *ART* covariates effects on the hazard are not constant over time and hence violated the constant covariate effect assumption. Since main effects *pred*, *ART*, and *gender* violated the assumption, we used the extended Cox‐PH model (Section 2.2) to estimate the effects of the covariates on the hazard of survival outcomes considered in this study.

### 3.3 Fitting the extended Cox‐PH model

For the *pred* variable under the composite outcome, top‐left panel of Figure 2, the time point where the hazard of the coefficient exceeds the reference for null effect is 43 and the point where the slope of the beta reverses and 59. These time points are used to divide the analysis time, into three groups (0–43, 43–59, and 59–800), using the *survSplit* function in *survminer* package. The same logic is used to divide analysis time under the Cox‐PH model for death, cardiac tamponade, and constriction. We now fit the extended Cox‐PH model and assess the constant covariate assumption. These results are presented in Table 2 confirmed that the all the covariates now meet the constant covariates effect over time. Tables 3 and 4 present the unadjusted hazard ratio (unaHR) and adjusted hazard ratio (aHR) with their corresponding standard errors and 95% confidence intervals (95% CI) from the extended or time‐depended Cox‐PH model for composite outcome and death and cardiac tamponade and, constriction, respectively.

**Table 2:** Testing the assumption of the extended Cox‐PH model.

Composite outcome				Death		
Variable	*χ* ^2^	*df*	*p* Value	*χ* ^2^	*df*	*p* Value
Prednisolone	4.72e−05	1	>0.99	0.9852	1	0.32
*ART*	0.2090	1	0.65	0.1616	1	0.69
Prednisolone × ART	1.1600	1	0.28	0.0666	1	0.80
Age	0.0023	1	0.88	2.0903	1	0.15
*bonarvs*	0.2480	1	0.62	0.0231	1	0.88
*gender*	0.6020	1	0.44	1.5852	1	0.21
pred:strata(tgroup)	0.0275	2	0.87	–	–	–
ART:strata(tgroup)	–	–	–	1.6708	2	0.43
Global	12.300	8	0.14	9.7068	8	0.29

Cardiac tamponade					Constriction	
Prednisolone	0.4741	1	0.49	1.2268	1	0.27
*ART*	1.8949	1	0.17	0.3252	1	0.57
Prednisolone × ART	0.0685	1	0.79	0.0066	1	0.94
Age	0.1192	1	0.73	1.1461	1	0.28
*bonarvs*	1.7806	1	0.18	0.2126	1	0.65
*gender*	0.9158	1	0.34	1.6610	1	0.20
pred:strata(tgroup)	0.4741	2	0.79	3.3616	1	0.07
strata(tgroup):sex	–	–	–	1.8182	1	0.19
strata(tgroup):bonarvs	–	–	–	0.5096	1	0.48
Global	8.9416	8	0.35	12.2059	9	0.20

Abbreviations: ART, antiretroviral therapy; Cox‐PH, Cox‐proportional hazard.

Table 3 presents the results of the extended Cox‐PH model for time to first occurrence of composite outcome or death. The results showed that there is 69% significant reduction in hazard of the composite outcome among patients randomized to receive prednisolone relative to those who received placebo. Both the unaHR and aHR showed that patients who received ART at each visit during the study have reduced hazard of composite outcome compared with those who were never on ART during the study. However, the unaHR was not statistically significant.

**Table 3:** Unadjusted and adjusted hazard ratio from the extended Cox‐PH model for composite outcome and death.

Time to composite outcome	Unadjusted HR				Adjusted HR			
	HR	SE	95%CI	*p* Value	HR	SE	95%CI	*p* Value
*pred*								
Placebo (ref)	–	–	–	–	–	–	–	–
Prednisolone	0.31	0.26	(0.19,0.51)	<0.001	0.32	0.27	(0.19,0.54)	<0.001
*ART*								
Never on ART during the study (ref)	–	–	–	–	–	–	–	–
Ever on ART during the study	0.81	0.12	(0.65,1.02)	0.07	0.64	0.19	(0.46,0.92)	0.02
*Predart*								
Prednisolone–ART interaction (ref)	–	–	–	–	–	–	–	–
Prednisolone–ART interaction	0.63	0.31	(0.34,1.17)	0.15	0.96	0.23	(0.61,1.51)	0.86
*agegrp*								
Age ≤46 (ref)	–	–	–	–	–	–	–	–
Age ≥46	1.58	0.15	(1.17,2.17)	0.002	1.61	0.15	(1.19,2.17)	0.002
*bonarvs*								
Not on ART at study entry (ref)	–	–	–	–	–	–	–	–
On ART at study entry	1.28	0.14	(0.98,1.68)	0.072	1.76	0.17	(1.25,2.47)	<0.001
*gender*								
Female (ref)	–	–	–	–	–	–	–	–
Male	1.50	0.12	(1.19,1.89)	<0.001	1.56	0.12	(1.23,1.97)	<0.001

Time to death								
*pred*								
Placebo (ref)	–	–	–	–	–	–	–	–
Prednisolone	1.62	0.15	(1.21, 2.16)	<0.001	1.58	0.18	(1.11, 2.24)	0.011
*ART*								
Never on ART during the study (ref)	–	–	–	–	–	–	–	–
Ever on ART during the study	0.4	0.39	(0.19,0.85)	0.018	0.41	0.86	(0.08,2.26)	0.31
*agegrp*								
Age ≤46 (ref)	–	–	–	–	–	–	–	–
Age ≥46	2.00	0.18	(1.40,2.86)	<0.001	2.08	0.18	(1.45,2.98)	≤0.001
*bonarvs*								
Not on ART at study entry (ref)	–	–	–	–	–	–	–	–
On ART at study entry	0.26	0.73	(0.06,1.07)	0.06	1.19	0.25	(0.73,1.93)	0.49
*gender*								
Female (ref)	–	–	–	–	–	–	–	–
Male	1.55	0.15	(1.15,2.09)	0.004	1.59	0.15	(1.18,2.15)	0.002

Abbreviations: ART, antiretroviral therapy; CI, confidence interval; Cox‐PH, Cox‐proportional hazard; HR, hazard ratio.

Although, the results showed that prednisolone‐ART interaction reduces the hazard of the composite outcome, there was no significant prednisolone‐ART interaction effect for both the unaHR and aHR. We also found that, for both the unaHR and aHR, the hazard of the composite outcome increased significantly by approximately 58% among patients who were above 46 years relative to those who are 46 years and below. The aHR showed that there is approximately 1.76‐fold increase in the hazard of the composite outcome among patients who were on ART at the study entry relative to those were not on ART at study entry. Although the unaHR showed 1.28‐fold increased in the hazard of the composite outcome, this effect was not statistically significant. The hazard of the composite outcome significantly increased by 1.56‐fold among male patients relative to female patients for the aHR and 1.51‐fold increase for the unaHR.

The results showed an increased hazard death among patients who received prednisolone versus placebo. Patients who are ever on ART during the study have reduced risk of death relative to those who were never on ART during the study. This reduction was not significant for the aHR. The results revealed that prednisolone‐ART interaction reduces the hazard of death but statistically not significant for both the unaHR and aHR. There is approximately twofold increase in the hazard of death among patients who are above 46 years relative to those who are 46 years and below. For the unaHR, patients who were on ART at the study entry have approximately 74% reduced risk of death relative to those who were not on ART at the study entry and 19% insignificant increased risk of death for the aHR. The hazard of death significantly increases among male patients relative to female patients.

Table 4 presents that results of the Cox‐PH model fitted to time to cardiac tamponade or constriction. There is a significant reduced hazard of cardiac tamponade among patients randomized into the prednisolone arm relative to those in the placebo arm, for both the and the aHRs. We observed an increased hazard of cardiac tamponade among patients who were ever on ART during the study relative to those who were not on ART during the study, for both unaHR and aHR. The unaHR showed that prednisolone‐ART interaction reduces the hazard of cardiac tamponade and increased risk for the aHR but is not statistically significant in both cases. Patients who are above 46 years have increased risk of cardiac tamponade relative to those who are 46 years and below. However, this increase is not statistically significant. There is approximately twofold increase in the hazard of cardiac tamponade among patients who were on ART at the study entry relative to those who not on ART at the study entry. However, this increase was not statistically significant for the unHR. There is an increased hazard of cardiac tamponade among male participants relative to female participants. However, this increase is not statistically significant.

**Table 4:** Unadjusted and adjusted odds ratio from the extended Cox‐PH model for cardiac tamponade and constriction.

Time to cardiac tamponade	Unadjusted HR				Adjusted HR			
	HR	SE	95% CI	*p* Value	HR	SE	95% CI	*p* Value
*pred*								
Placebo (ref)	–	–	–	–	–	–	–	
Prednisolone	0.20	0.49	(0.07, 0.52)	<0.001	0.14	0.55	(0.05, 0.42)	<0.001
*ART*								
Never on ART during the study (ref)	–	–	–	–	–	–	–	
Ever on ART during the study	1.94	0.28	(1.11, 3.37)	0.02	1.315	0.45	(0.55, 3.17)	0.54
*Predart*								
No prednisolone–ART interaction (ref)	–	–	–	–	–	–	–	
Prednisolone–ART interaction	0.69	0.55	(0.24, 2.02)	0.50	1.12	0.57	(0.37, 3.41)	0.84
*agegrp*								
Age ≤ 46 (ref)	–	–	–	–	–	–	–	
Age ≥ 46	1.15	0.41	(0.52, 2.54)	0.73	1.06	0.41	(0.48, 2.35)	0.89
*bonarvs*								
Not on ART at study entry (ref)	–	–	–	–	–	–	–	
On ART at study entry	1.56	0.23	(0.99, 2.43)	0.05	2.03	0.35	(1.02, 4.03)	0.04
*gender*								
Female (ref)	–	–	–	–	–	–	–	
Male	1.17	0.28	(0.67, 2.03)	0.58	1.28	0.28	(0.73, 2.22)	0.39

Time to constriction								
*pred*								
Placebo (ref)	–	–	–	–	–	–	–	
Prednisolone	0.37	0.23	(0.23, 0.57)	< 0.001	0.81	0.35	(0.41, 1.61)	0.55
*ART*								
Never on ART during the study (ref)	–	–	–	–	–	–	–	
Ever on ART during the study	0.94	0.20	(0.63, 1.39)	0.76	0.68	0.29	(0.38, 1.21)	0.19
*Predart*								
Noprednisolone–ART interaction (ref)	–	–	–	–	–	–	–	
Prednisolone–ART interaction	0.38	0.33	(0.20, 0.74)	0.004	0.86	0.48	(0.34, 2.21)	0.76
*agegrp*								
Age ≤ 46 (ref)	–	–	–	–	–	–	–	
Age ≥ 46	1.06	0.31	(0.58, 1.94)	0.85	1.14	0.31	(0.62,2.10)	0.67
*bonarvs*								
Not on ART at study entry (ref)	–	–	–	–	–	–	–	
On ART at study entry	1.56	0.23	(0.99, 2.44)	0.05	1.20	0.44	(0.51, 2.83)	0.68
*gender*								
Female (ref)	–	–	–	–	–	–	–	
Male	1.17	0.28	(0.67, 2.03)	0.58	0.86	0.30	(0.48, 1.54)	0.61

Abbreviations: ART, antiretroviral therapy; CI, confidence interval; Cox‐PH, Cox‐proportional hazard; HR, hazard ratio; SE, standard error.

The results revealed that prednisolone has significant effect in reducing the hazard of constriction. Patients who were ever on ART during the study is associated with a reduced risk of constriction relative to those who were never on ART during the study. Prednisolone‐ART interaction has no significant effect on the hazard of constriction. We found that age has no significant effect on the hazard of constriction though there is an increase in the hazard of constriction among patients who are above 46 years relative to those who are 46 years and below. We observed an insignificant increased hazard of constriction among patients who were on ART at the study entry relative to those who were not on ART at the study entry. Gender has no significant on the hazard of constriction though there is an increased hazard among male subject when compared with female subjects.

## 4 DISCUSSION

This paper performed analyses to investigate the effects of prednisolone as well as prednisolone–ART interaction on time‐to‐first occurrence of the composite outcome, death, cardiac tamponade, and constriction among TBP and only HIV‐positive patients in the IMPI trial.^10^, ^11^ This main objective was achieved by using the Cox‐PH model. This study also adjusted for the effects of covariates such as age, gender, whether patient was on ART at study entry, patient was ever on ART during the study.

We fitted the Cox‐PH model to survival outcomes, adjusting for potential risk factors of the hazard of events, using R version 4.3.1. We estimated parameters from the Cox‐PH model using *coxph* function from *survival* package.^45^, ^46^ Because of the covariates in the Cox‐PH models for the survival outcomes violated the constant covariate effect over time assumption, we fitted the extended or time‐dependent Cox‐PH model. In this model, the continuous analysis time was stratified into groups of time intervals by identifying time points at which such covariate makes a major change along the analysis time by observing the plots of the Schoenfield residuals versus the analysis time. Using the identified time points, the analysis time is then stratified into group of time intervals by using the *survSplit* function in *survminer* package.

The results showed that prednisolone reduces the hazard of the composite outcome, indicating that prednisolone have protective effect against the composite outcome. ART usage is associated with a lower risk of the composite outcome, where the unaHR did not reach statistical significance. This discrepancy between the unaHR and aHR may be due to the influence of other covariates or factors that were accounted for in the adjusted analysis. There is no significant prednisolone–ART interaction. The study reveals a significant increase in the hazard of the composite outcome among patients who are above 46 years compared to those who are 46 years and below. Patients who were on ART at the study entry exhibited a notable 1.76‐fold increase in the hazard of the composite outcome according to the aHR. There is an increased hazard of composite outcome among male patients relative to female subjects. This gender disparity may be attributed to various factors, including biological differences or behavioral patterns.

There is an increased hazard of death among patients who received prednisolone compared to those who received a placebo. It is essential to interpret this result with caution since the main causes of death, in the IMPI trial were pericarditis (23.8%), disseminated TB (18.6%), HIV infection (7.3%), and other cardiovascular causes (5.7%).^11^ This effect is significant in this study because the study considered only HIV‐positive patients. The results showed a significant twofold increase in the hazard of death among patients who are above 46 years relative to those who are 46 years and below. This highlights the critical role of age as a risk factor for mortality in the study population, with older individuals facing a significantly higher risk of death. The hazard of death significantly increases among male patients compared to female patients. This gender disparity highlights the importance of considering gender‐specific factors that may contribute to differences in mortality risk.

Prednisolone‐randomized patients had a significant reduction in the hazard of cardiac tamponade compared to those in the placebo arm. We observed a significant effect of prednisolone in reducing the hazard of constriction, a finding which agrees with that from Mayosi et al.^11^ and Gumedze et al.^47^ study revealed that HIV‐infected TBP patients were less likely to develop constrictive pericarditis. Patients who were ever on ART during the study showed a reduced risk of constriction relative to those who were never on ART.

## 5 CONCLUSION

Prednisolone is associated with a significant reduction in the hazard of the composite outcome, cardiac tamponade and constriction and consistent ART usage is associated with a reduced risk of these outcomes. These findings contribute to our understanding of the impact of prednisolone and ART on the outcomes in the context of the study population. The study provides valuable insights into the effects of prednisolone and ART on the hazard of various outcomes in TBP and HIV‐positive patients. The findings have potential clinical implications, especially in informing treatment decisions and strategies to improve outcomes in this patient population. The protective effect of prednisolone against the outcomes is noteworthy, despite the concern regarding increased risk of death associated with prednisolone.

The clinical implications of the study suggest that the administration of prednisolone yields a protective effect against the composite outcome, as well as specific complications such as cardiac tamponade and constriction, particularly in patients diagnosed with both TBP and HIV. This finding underscores the potential therapeutic benefit of incorporating prednisolone into the treatment regimen for this patient population, with the potential to improve overall clinical outcomes and mitigate adverse cardiac events. However, further research and clinical trials are warranted to validate and refine these implications before widespread implementation in clinical practice. The IMP trial was a cardiology trFor future cardiology trials with TB patients, equal consideration should be accorded to HIV‐related data, such as CD4 count, to ensure a comprehensive evaluation of its impact on the primary outcome.

The study, as indicated by Mayosi et al.,^11^ has few limitations. First, only a quarter of the patients received a definite diagnosis of TB, either in the pericardium or elsewhere in the body. Consequently, one potential interpretation of the trial results, encompassing both HIV‐negative, HIV‐positive, and TBP patients, could suggest that the interventions were ineffective due to the relatively low proportion of trial participants diagnosed with TBP. Nonetheless, the consistency of results between patients with definite and probable TB suggests that prednisolone is anticipated to confer a protective effect against the composite outcome, cardiac tamponade, and constriction, as demonstrated in this study, specifically for HIV‐positive and TBP patients. Additionally, the challenging nature of diagnosing extrapulmonary TB is acknowledged, with only a minority of extrapulmonary TB cases being treated based on a definite diagnosis.^48^

The second limitation pertains to a minor fraction of patients (less than 2%) who received a diagnosis other than TB. Notwithstanding that the determination of the study's required sample size relied on the clinical case definition of TBP,^4^ it was anticipated that a small percentage of cases (up to 10%) might be attributed to an alternative cause of pericarditis.^10^ The third limitation concerns the trial's power calculation, which was based on an anticipated nonadherence rate of 10% in the active‐treatment groups (prednisolone and *M. indicus pranii*). While the prednisolone group nearly met this target with a nonadherence rate of 11%, the *M. indicus pranii* group experienced a higher nonadherence rate (21%), primarily attributed to injection‐site side effects.^11^ This relatively elevated nonadherence rate might have compromised the study's statistical power, particularly in the analysis of the primary outcome within the *M. indicus pranii* group.

Finally, given that prednisolone possesses immunosuppressive properties while M. indicus pranii is immunostimulatory, there is a possibility of interaction between them, potentially leading to either a reduction or enhancement of each other's effects. Nevertheless, multiple authors^11^, ^21^, ^23^, ^49^ have demonstrated that prednisolone and M. indicus pranii do not interact. As a result, trial arms can be analyzed separately along with their respective placebo arms.

## AUTHOR CONTRIBUTIONS

**Abdul‐Karim Iddrisu**: Conceptualization; data curation; formal analysis; methodology; software; supervision; validation; visualization; writing—original draft; writing—review and editing. **Dominic Otoo**: Methodology; software; writing—original draft; writing—review and editing. **Afa Kwasi**: Methodology; software; writing—original draft; writing—review and editing. **Freedom Gumedze**: Conceptualization; data curation; methodology; supervision.

## CONFLICT OF INTEREST STATEMENT

The authors declare no conflict of interest.

## TRANSPARENCY STATEMENT

The lead author Abdul‐Karim Iddrisu affirms that this manuscript is an honest, accurate, and transparent account of the study being reported; that no important aspects of the study have been omitted; and that any discrepancies from the study as planned (and, if relevant, registered) have been explained.

## Data Availability

Authors do not have the right to distribute data.
